# Childhood disability in rural Niger: a population-based assessment using the Key Informant Method

**DOI:** 10.1186/s12887-022-03226-0

**Published:** 2022-03-31

**Authors:** Lena Morgon Banks, Jing Liu, Anne Kielland, Ali Bako Tahirou, Abdoul Karim Seydou Harouna, Islay Mactaggart, Ragnhild Dybdahl, Dan Firoun Mounkaila, Arne Grønningsæter

**Affiliations:** 1 International Centre for Evidence in Disability, London School of Hygiene & Tropical Medicine, London, UK; 2 Fafo, Oslo, Norway; 3 Laboratoire d’Études Et de Recherche Sur Les Dynamiques Sociales Et Le Développement Local (LASDEL), Niamey, Niger; 4 Norwegian National Institute for Health, Oslo, Norway; 5 Fédération Nigérienne Des Personnes Handicapées, Niamey, Niger

## Abstract

**Background:**

Data on childhood disability is essential for planning health, education and other services. However, information is lacking in many low- and middle-income countries, including Niger. This study uses the Key Informant Method, an innovative and cost-effective strategy for generating population-based estimates of childhood disability, to estimate the prevalence and causes of moderate/severe impairments and disabling health conditions in children of school-going age (7–16 years) in the Kollo department of western Niger.

**Methods:**

Community-based key informants were trained to identify children who were suspected of having the impairment types/health conditions included in this study. Children identified by key informants were visited by paediatricians and underwent an assessment for moderate/severe vision, hearing, physical and intellectual impairments, as well as epilepsy, albinism and emotional distress.

**Results:**

Two thousand, five hundred sixty-one children were identified by key informants, of whom 2191 were visited by paediatricians (response rate = 85.6%). Overall, 597 children were determined to have an impairment/health condition, giving a prevalence of disability of 11.4 per 1000 children (10.6- 12.2). Intellectual impairment was most common (6.5 per 1000), followed by physical (4.9 per 1000) and hearing impairments (4.7 per 1000). Many children had never sought medical attention for their impairment/health condition, with health seeking ranging from 40.0% of children with visual impairment to 67.2% for children with physical impairments.

**Conclusion:**

The Key Informant Method enabled the identification of a large number of children with disabling impairments and health conditions in rural Niger, many of whom have unmet needs for health and other services.

**Supplementary Information:**

The online version contains supplementary material available at 10.1186/s12887-022-03226-0.

## Introduction

Robust estimates on the prevalence of childhood disability are lacking, particularly in low- and middle-income countries [1]. It is widely reported that 5% of children globally are living with moderate to severe disabilities [2]. However, the data underlying this estimate stems from “inconsistent, fragmented and partial data” arising from the 2004 Global Burden of Disease update [1, 3].

Disability is defined by the United Nations Convention on the Rights of Persons with Disabilities (UNCRPD) as including “those who have long-term physical, mental, intellectual or sensory impairments which in interaction with various barriers may hinder their full and effective participation in society on an equal basis with others” [4]. Closely linked to this definition is the International Classification of Functioning, Disability and Health’s (ICF) model of disability. Under the ICF, individuals can have a health condition that causes an impairment in one or more body structures or functions [5]. Impairments in turn can lead to activity limitations (e.g. difficulty walking, remembering) or participation restrictions (e.g. going to school, work), depending on their interaction with personal or environmental contextual factors (e.g. their access to assistive devices, social attitudes towards disability, accessibility of infrastructure and communication) [5]. For example, two children may have the same level of visual impairment, but different levels of functioning and participation based on if they have access to glasses or other assistive devices and the extent to which their environment is disability-inclusive (e.g. inclusive education options are available and affordable, attitudes about disability in their family and community).

Good quality data on childhood impairments and disability is needed to inform national and sub-national policy and planning. For example, data on the prevalence of disability and impairments are essential for designing and budgeting for health services, inclusive education and community-based programmes. The importance of data on disability is increasingly recognised in national and international commitments. For example, Article 31 of the UNCRPD obliges the 184 ratifying States to collect data on disability to monitor and formulate appropriate policies to support the implementation of the UNCRPD [6]. Further, the 2030 Sustainable Development Goals (SDGs) calls for the collection of data on disability for disaggregation of all goals and indicators by disability status to ensure its mandate of “no one left behind” from development progress [7].

The Key Informant Method (KIM) is an innovative strategy used to identify target groups for research or programme delivery, particularly groups with relatively low prevalence in community settings (e.g. sex workers, people living with HIV, people with mental health conditions) [8–10]. The KIM makes use of community-based volunteers (Key Informants) with strong standing within and knowledge of their communities, who, with appropriate training, can identify target individuals at less cost than a standard population-based survey [8]. The KIM has been used and validated for identifying and measuring the prevalence of childhood disability in several settings [8, 11]. For example, it has been used to identify children with sensory, physical and intellectual impairments and epilepsy in Malawi [8], Kenya [12], India [13] and Bangladesh [14]. The KIM trains community-based volunteers to identify children with potentially disabling impairments, who are then examined by medical professionals. The KIM is considered valid and cost-effective compared to other methods for estimating prevalence of impairments in children [8]. For example, a study in Bangladesh estimated that the KIM cost £90 per child identified with an impairment, compared to £352 per child for a population-based survey in the same setting [14].

The aim of this study is to estimate the prevalence of disability in children of school-going age (7–16 years) in the Kollo department of the Tillabéri region of west Niger using the KIM. It also assesses the type and aetiology of disability, as well as health-seeking behaviours. Niger is a low-income country, which ranks at the bottom of the Human Development Index [15, 16]. Previous estimates of disability prevalence from the 2012 census indicate that 3.7% of boys and 3.8% of girls have a disability [17]. Prevalence of disability was higher in rural areas of Niger compared to urban areas (0.1–0.6% amongst children 5–14 years in urban settings vs 2.4–2.8% in rural areas) [17], which follows global trends [2]. However, disability in the census used only a single question, which is not recommended for providing robust prevalence estimates [18]. Further, data on clinical impairments and aetiology is important for informing the provision of services.

## Methods

### Study setting and population

This study was conducted in the Kollo department of the Tillabéri region of west Niger. Kollo is predominantly rural, agrarian area, with a population of 465 399 as of the 2012 census [19]. The all-age prevalence of disability in Kollo was estimated at 3.6% in 2012 [19]. The literacy rate is 26.6% and school attendance rate is 68.3% in Kollo, which is slightly higher than the regional and national averages [19–21]. Over 50% of the population is considered poor, and approximately 40–60% of young children experience stunting [22]. Services such as clean water and sanitation and health services are generally in short supply and of poor quality [19, 22]. Kollo was selected as the study site due to its proximity to the capital Niamey and its relative safety compared to other areas of Niger at the time of data collection.

The study population was community-dwelling children ages 7–16 years old living in the communes of Kouré, N’Dounga, Kirtachi, Hamdallaye and Diantchandou within Kollo department. This age group was selected to reflect the standard school-going ages for Niger (primary and lower secondary) as this study was embedded within a larger project on access to education (“Addressing Local Barriers to Inclusive Education for Children with a Disability in the Sahel”, Research Council of Norway case number 759423). The underlying population in our study area was estimated at 68,844 children ages 7–16 years, based on the 2012 census and updated to reflect population growth.

### Data collection

Data was collected between September 2018-March 2019. Children were screened and assessed for disability through a two-part process, first by key informants and then through a clinical assessment carried out by paediatricians. Details on the KIM process have been described in detail elsewhere [11], but are summarised in brief below.

#### Preliminary identification by Key Informants

Each of the five communes had a Commune Facilitator who was responsible for coordinating with village chiefs to select Key Informants (KIs). In total, 185 villages (and surrounding areas) were identified, each of which was assigned one or more KIs (187 KIs total) to identity children with specified impairments and health conditions (approximately 370 children in the target age range per KI). KIs were selected based on their familiarity with and acceptance in their designated village, with many serving in trusted roles (e.g. local government, teachers). KIs also required sufficient written literacy skills to complete basic listing forms of identified children (i.e. recording name, address). The KIs and Commune Facilitators attended a one-day training workshop in September 2018 which covered: background and sensitisation on disability; the identification of specific impairments and health conditions relevant to this study (e.g. visible indicators and behavioural signs associated with each impairment type); methods for case finding and procedures for listing children; and practice exercises to test KI learning. Trainings were provided at a central location in each of the 5 communes by an expert in disability research (LMB) who has worked on previous KIM studies and a Nigerien researcher (AB) who has worked extensively on social science studies in the setting.

In the two months following training, KIs identified and listed children suspected to have the impairments and health conditions specified in the training, with oversight from the Commune Facilitator. The specified impairments and health conditions that KIs were trained to identify for were visual, hearing, physical and intellectual impairments, as well as epilepsy, albinism and emotional distress. To identify as many children as possible in their assigned area, KIs were instructed to: a) spread information about the study in the community so that caregivers and other community members could come forward about children with suspected impairments/health conditions; b) liaise with community leaders to compile a list of children believed to have the specified impairments; and c) visit households to enquire about children in the target age range. If KIs determined the child likely had an eligible impairment type/health condition and was within the study age range (7–16 years), their details (e.g. child/caregiver names, approximate address) were recorded in a listing form so that they could be revisited by paediatricians. KIs were instructed to be non-conservative in including children in the listing when in doubt.

#### Assessment by paediatricians

Six paediatricians visited the children listed by KIs to provide an assessment of disability. Paediatricians received a five-day training on study protocols, including the use of mobile applications used for clinical screening of impairments (e.g. PeekAcuity for visual acuity, HearTest for auditory). Previous KIM studies have invited children identified by KIs to attend screening camps where they are examined by health professionals. However, non-attendance rates have been high in previous studies (e.g. 48% in Malawi, 52% in Bangladesh) due to accessibility, financial and informational challenges [8, 23]. Consequently, doctors visited children in their homes or other local meeting point to minimise attrition.

Children identified by KIs as having potentially disabling impairments underwent an assessment of disability, which involved two stages. In the first stage, guardians and/or children answered questions from the Washington Group-UNICEF module on Child Functioning (ages 5–17) [24]. The Washington Group-UNICEF module asks about the child’s level of difficulty in performing everyday activities, with any assistive devices (e.g. hearing aids, glasses) if currently used by the child. Most questions have four possible responses: no difficulty, some difficulty, a lot of difficulty or cannot do. Additional questions on whether the child had albinism or had experienced seizures were also added to the initial screening. These health conditions were added based on feedback from Disabled People’s Organisations (DPO) in Niger on local conceptualisations of disability and from similar KIM studies, which found these health conditions are often disabling [8]. Children aged 10 years and older self-reported on their level of functioning, while the primary guardian answered on behalf of children younger than 10 years, and for children with severe difficulties understanding/communicating.

In the second stage, doctors conducted relevant clinical assessments based on the child’s answers to the Washington Group-UNICEF screening questionnaire. The mapping of Washington Group-UNICEF screening questions to clinical assessment domains, as well as the cut-offs for determining the presence of a clinical impairment/disabling health condition are described in Table 1.

**Table 1:** Clinical assessment method and definitions of impairment

Impairment/health condition	Washington Group screening triggers	Screen/Exam	Assessment	Case definition
Moderate/severe vision impairment	At least some difficulty seeing (with glasses if used), learning	Exam	Visual acuity test using PeekAcuity. Eye exam with direct ophthalmoscope by paediatrician	logMar ≥ 0.5 for both eyes
Moderate/severe hearing impairment	At least some difficulty hearing (with hearing aids if used), being understood by people inside or outside of the household, learning	Exam	Pure Tone Audiometry (PTA) using HearTest; ear exam by paediatrician using an otoscope	≥ 31 dB for both ears
Moderate/severe physical impairment	At least some difficulty walking 100 m or 500 m, with equipment if used, with self-care, being understood by people inside or outside of the household, self-care	Exam	Standardised observation of activities (ability to hold and change position, mobility, and hand function)	Cannot do at least one activity, with functional difficulty reported to have lasted at least one month
Epilepsy	(Non-Washington group): Child has ever had a seizure	Screen	Four screening questions on type and frequency of epilepsy episodes in the last year	Reported three or more seizures in the last year
Albinism	(Non-Washington Group): Caregiver report/visual confirmation	n/a	n/a	Caregiver report, visual confirmation
Intellectual impairment	At least some difficulty with self-care, being understood by people inside or outside of the household, learning, remembering, concentrating on an enjoyed activity, accepting changes to his/her routine, controlling his/her behaviour, making friends, self-care	Screen	12 age-relevant questions on communication, behaviour, comprehension, concentration, relationships and learning^a^	Screens positive on at least 3 questions
Emotional distress	At least some difficulty being understood by people inside or outside of the household, learning, remembering, concentrating on an enjoyed activity, accepting changes to his/her routine, controlling his/her behaviour, making friends; seems anxious, nervous or worried daily or weekly; seems sad or depressed daily or weekly	Screen	PHQ-A (depression) [25] CRIES-8 (PTSD) [26, 27]	Depression (PHQ-A): score ≥ 10 PTSD: score of ≥ 17

^a^This question set was developed and used in a KIM in Malawi [8] and was reviewed for relevance to Niger by Nigerien paediatricians

This assessment of disability combined information on both the child’s functioning (Washington Group-UNICEF module) and on clinical impairments (paediatrician assessment). This combination reflects a definition of disability that is more in line with the UNCRPD and the ICF, which views disability as arising from restrictions in functioning and participation due to the interaction of a clinical impairment with personal and environmental barriers [4, 28].

Finally, caregivers of children who were found to have a clinical impairment were asked questions about the attributed cause of the impairment and health-seeking behaviour using a short closed-ended survey adapted from previous research [29].

### Data analysis

Data was analysed using R 3.5.3 [30]. The denominator for overall and impairment-specific prevalence estimates was the total number of children (7–16 years) living in the study area (*n* = 68,844). This figure was calculated using data from the 2012 census, which was adjusted to reflect population growth using United Nations Population Projections and data from the Niger Bureau of Statistics [31–33]. Overall and impairment-specific prevalence estimates were also adjusted to account for non-response in children identified by KIs, by assuming that prevalence (both overall and by impairment type) was the same as in children who were successfully followed up by paediatricians and able to complete the relevant clinical assessments. Further, one village in the sampled area was used as a pilot, and so prevalence was similarly adjusted assuming the same proportion as in the rest of the study areas. Finally, some children who were followed up by paediatricians were unable to complete all relevant assessments (e.g. for hearing, vision), and so prevalence was adjusted to reflect the prevalence of impairments in children who did complete the assessments and screened positive to similar Washington Group questions triggering the uncompleted assessment. This method of estimating prevalence has been used in other KIMs [8].

Sensitivity analysis was performed by varying the overall and impairment-specific prevalence in non-response and pilot children by ± 10% of the prevalence in children followed up, which has been used in other studies [8, 34]. All prevalence figures include 95% confidence intervals (95% CIs).

### Ethical considerations

The study received ethical approval from the Comité National Éthique pour la Recherche en Santé [National Ethics Committee for Health Research] in Niger. The study was also registered with the Norwegian Centre for Research Data, which assessed that the processing of personal data in this project is in accordance with data protection legislation. The study purpose and procedures were explained to children and their guardians prior to starting any assessments. Informed consent was obtained from the guardians of all participating children, as they were all below the national age of consent (18 years). However, children provided their assent to participate. Respondents were not provided with any remuneration (in cash or kind) for their participation. Guardians were informed about the results of their child’s clinical assessment, and children with unmet health needs were referred for further services by the examining clinician where possible. Study results were presented to key stakeholders, including members of Organisations of Persons with Disabilities, in the capital Niamey in January 2020.

## Results

**Fig. 1:**
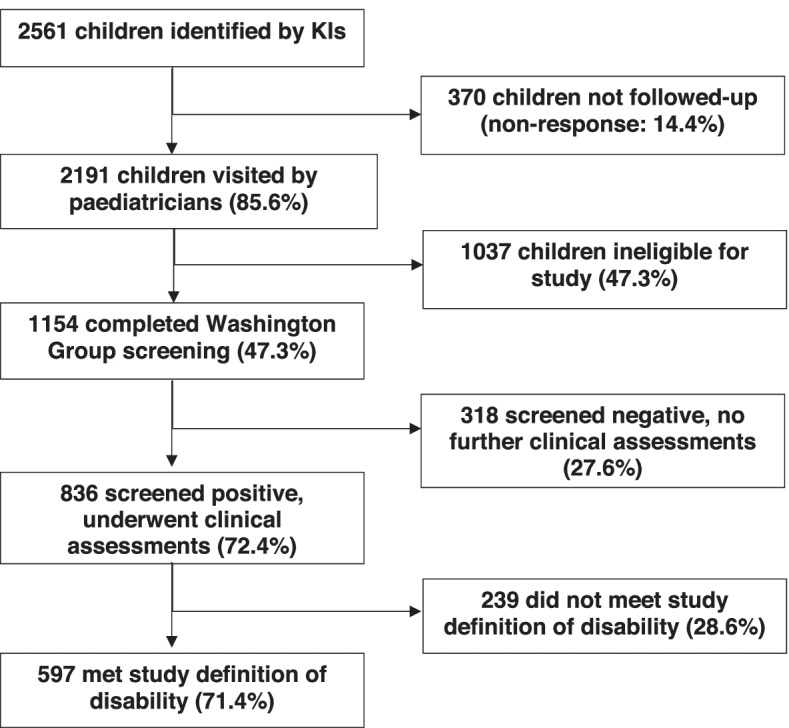
Flow chart of children identified and assessed

In total, 2,561 children were listed by KIs as potentially having an impairment, of whom 2,191 were visited by the paediatricians (response rate: 85.6%) (Fig. 1). Children not followed up were slightly older (11.5 vs 10.6 years, *p* < 0.001) and more likely to be male (66% vs 59%, *p* = 0.01); there was no difference in response rate by commune. Of the 2,191 children followed-up, 1,154 (52.7%) were eligible for this study and screened for disability. The main reasons children were ineligible were being outside the study age range, or their presenting concern was non-disabling (e.g. skin rash). The 1154 children were first screened using the Washington Group questions, of whom 836 (72.4%) scored positive in at least one domain (16.0/1000, 95% CI: 15.1–16.9) and were eligible for further clinical assessment. Of the 836 children clinically assessed, 597 (71.4%) met the study definition of disability.

### Prevalence of disability and specific impairments/health conditions

Overall, 597 children met the study definition of disability, giving an estimated prevalence in the study area of 11.4 children per 1000 (95% CI: 10.6–12.2) (Table 2). The most common impairment type/health condition was intellectual impairments (6.5/1000, 95% CI: 5.9–7.1), followed by physical (4.9/1000, 95% CI: 4.4–5.4) and hearing (4.7/1000, 95% CI: 4.2–5.2) impairments. Almost half of children with a disability had multiple impairments (4.5/1000, 95% CI: 4.0–5.0). Prevalence of disability was slightly higher for boys (12.9/1000, 95% CI: 11.7–14.1) compared to girls (9.9/1000, 95%CI: 8.9–10.9). Epilepsy, hearing and intellectual impairments were more common amongst boys than girls (Supplemental Table 1).

**Table 2:** Prevalence estimates of impairments/health conditions in children in the study area

**Impairment/health condition**	**Number**	**Prevalence per 1000 (95% CI)**^a^	**Prevalence per 1000 range**^**b**^
Disability (any impairment/health condition)	597	11.4 (10.6- 12.2)	11.2 – 11.7
Physical impairment	253	4.9 (4.4—5.4)	4.7 – 5,0
Hearing impairment	133	4.7 (4.2—5.2)	4.6—4.8
Visual impairment	45	1.2 (0.9—1.5)	1.2—1.2
Intellectual impairment	338	6.5 (5.9—7.1)	6.3—6.6
PTSD/Emotional distress	31	0.6 (0.4—0.8)	0.6—0.6
Epilepsy	100	1.9 (1.6 – 2.2)	1.9 – 2,0
Albinism	7	0.1 (0.0—0.2)	0.1—0.1
Multiple	236	4.5 (4.0 – 5.0)	4.4—4.6

^a^Based on assumption that prevalence of disability and impairments/health conditions was the same in children in the pilot village and in non-responders (overall and for specific screens)

^b^Based on sensitivity analysis assuming the prevalence in the non-responders, pilot village was ± 10% of prevalence in children followed-up

These figures were based on the assumption that non-responders (i.e. children listed by KIs but not seen by paediatricians) and children in the pilot village had the same prevalence of disability and specific impairments/health conditions as children who were followed-up and assessed. Sensitivity analyses varied the prevalence in non-responders/pilot village children by ± 10% of prevalence in children followed-up, which gave similar estimates of prevalence as with the original assumption of no difference in prevalence.

#### Physical impairments

Overall, 518 diagnoses were provided for the 253 children with physical impairments (Supplemental Table 2). A neurological diagnosis was most common (*n* = 206, 39.8%), followed by congenital (*n* = 184, 35.5%), acquired non-traumatic (*n* = 90, 17.4%) and traumatic (*n* = 38, 7.3%). The most common diagnoses listed by paediatricians were of cerebral palsy (*n* = 26), unspecified developmental delay (*n* = 33), hemiplegia (*n* = 32) and epilepsy (*n* = 61). For 73 (14.1%) instances, the paediatricians were unable to provide a specific diagnosis of a health condition.

#### Hearing impairments

In total, 133 children (4.7 per 1000, 95% CI: 4.2—5.2) had bilateral hearing impairments. Most children identified had a profound hearing impairment (81 + dB; 66.2%) (Table 3). The most common diagnoses were sensorineural congenital (32.3%), sensorineural acquired (25.6%) and serous otitis media (4.5%). Paediatricians were unable to provide a diagnosis for 16.5% of cases.

**Table 3:** Severity and causes of hearing impairment

**Severity**	**# of children**	**%**
Moderate (31–60 dB)	29	21.8
Severe (61–80 dB)	12	9
Profound (deaf) (81 + dB)	92	69.2
**Cause**	**# of diagnoses**^**a**^	**%**
Sensorineural congenital	86	30.7
Sensorineural acquired	68	24.3
Impact ear wax	4	1.4
Foreign Body	1	0.4
Otitis Externa	2	0.7
Acute Otitis Media	8	2.9
Chronic Otitis Media	45	16.1
Serous Otitis Media	12	4.3
Other	10	3.6
Cause not given	44	15.7

^a^Diagnoses are by ear; multiple diagnoses permitted

The Washington Group screening triggered a hearing assessment for 453 children, which was completed by only 231 children. Children who did not complete the hearing assessment often had intellectual impairments (*n* = 202, 91% of non-completed).

### Visual impairments

In total, 45 children had bilateral visual impairment (1.2 per 1000, 95% CI: 0.9–1.5). Most assessed children had profound visual impairment (logMar > 1.3, 56.6%) (Table 4). Cataract was the most common cause (33.9%). In 22.0% of cases, the paediatrician was unable to provide a diagnosis.

**Table 4:** Severity and causes of visual impairment

**Severity (logMar)**	**N of children**	**%**
Moderate (≥ 0.5 and ≤ 1)	17	37.8
Severe (> 1 and ≤ 1.3)	0	0
Profound (blind) (> 1.3)	28	62.2
**Causes**	**N of diagnosis**^**a**^	**%**
Refractive Error	11	12.2
Cataract	30	33.3
Aphakia	2	2.2
Surgical	1	1.1
Other Corneal Scar	2	2.2
Globe Abnormality	2	2.2
Cortical Blind	12	13.3
Other	10	11.1
Unknown	20	22.2

^a^Multiple diagnoses permitted

452 children were identified during the screening as needing to take the vision assessment, but only 260 completed the assessment. Children who did not complete the vision assessment often had intellectual impairments (*n* = 176, 92% of children not completing vision assessment).

#### Intellectual impairments

Intellectual impairments were the most prevalent type of impairment/health condition assessed, with a prevalence of 6.5 children per 1000 (95% CI: 5.9–7.1). In most cases, paediatricians could not provide a specific diagnosis of the underlying health condition and all diagnoses provided were based on their professional opinion rather than by formal assessment. The most common types of diagnoses for intellectual impairment included conditions linked to cerebral palsy (*n* = 40, 11.8%), Down syndrome (*n* = 6, 1.8%), or linked to hypoxia from epilepsy (*n* = 22, 6.5%).

#### Epilepsy, albinism and emotional distress

Prevalence of epilepsy and albinism were 1.9 per 1000 children (95% CI:1.6 – 2.2) and 0.1 per 1000 children (95% CI: 0.0—0.2), respectively. Prevalence of emotional distress (symptoms of depression and post-traumatic stress) was 0.6 per 1000 children (95% CI: 0.4–0.8).

#### Multiple impairments

Overall, 236 (39.5%) of children with disabilities had multiple impairments. Amongst children with multiple impairments, 69.9% had two impairments, 22.5% had 3 impairments and 7.6% had 4 or more impairments. Children with emotional distress were most likely to have other impairments (83.9%), followed by children with epilepsy (69.0%).

### Attributed causes and health seeking

Caregivers of children with disabilities primarily attributed their child’s impairment/health condition to biomedical causes (e.g. genetic, illness, trauma), although slightly over 10% felt their child’s impairment was due to supernatural causes (Table 5). Many children had never been to see a health professional for their impairment/health condition, with health seeking ranging from 67.2% for children with physical impairments to 40.0% for children with visual impairments. For children for whom medical advice had been sought, less than a third of caregivers found that the services received were helpful. The most common reason for not seeking health services was due to a belief that the services would not be helpful, which was reported by approximately two-thirds of caregivers. The second most common reason across impairment types was due to prohibitive costs.

**Table 5:** Attributed causes of disability and health-seeking behaviours

	Emotional (*n* = 31)		Physical (*n* = 253)		Visual (*n* = 43)		Hearing (*n* = 133)		Cognitive (*n* = 338)		Epilepsy (*n* = 64)	
	n	%	n	%	n	%	n	%	n	%	n	%
Attributed causes												
Genetic/born this way	10	32.3	103	40.7	20	46.5	30	22.6	171	50.6	16	25.0
From trauma/ injury	3	9.7	19	7.5	0	0	0	0	9	2.7	2	3.1
From illness	12	38.7	97	38.3	10	23.3	74	55.6	110	32.5	32	50.0
Act of God/ supernatural	4	12.9	28	11.1	8	18.6	19	14.3	41	12.1	12	18.8
Other	0	0	2	0.8	0	0	4	3	0	0	0	0
Unknown	2	6.5	4	1.6	5	11.6	6	4.5	7	2.1	2	3.1
Health seeking												
Has seen medical doctor	19	61.3	170	67.2	18	40.0	55	41.4	155	45.9	45	45.0
Medical services helpful^a^	5	26.3	53	31.2	4	22.2	20	36.4	40	25.8	19	42.2
Reasons for not seeking medical advice^b^												
Do not need/Not useful	8	66.7	60	72.3	15	0.6	54	69.2	132	72.1	16	84.2
Too expensive	0	0	28	33.7	10	0.4	25	32.1	62	33.9	4	21.1
Services not available	1	8.3	1	1.2	0	0	2	2.6	4	2.2	0	0
Too far/no transportation	0	0	13	15.7	1	0	5	6.4	20	10.9	3	15.8
No time	0	0	1	1.2	0	0	1	1.3	2	1.1	0	0
Do not know where to go	2	16.7	6	7.2	2	0.1	3	3.8	20	10.9	2	10.5
Other	1	8.3	1	1.2	0	0	1	1.3	2	1.1	0	0

^*a*^*Amongst those who had sought services: *^*b*^*Amongst those who had not sought medical advice*

## Discussion

This is the first KIM used to estimate the prevalence and causes of disability in western Africa. Overall, 597 children were identified as having an eligible impairment/health condition, giving a prevalence of disability of 11.4 per 1000 (95% CI: 10.6–12.2) amongst children 7–16 years. This study captured data on both impairments (clinical assessments) as well as functioning (Washington Group-UNICEF Child Module screening), which is in line with the definition of disability described in the ICF.

The prevalence estimates found in this area of Niger are similar to other KIM studies. For example, prevalence of disability in children under 18 was 17.3 per 1000 in Malawi (95% CI: 16.9–17.7 per 1000) and 7.5 per 1000 (95% CI: 6.6–8.3 per 1000) for children under 10 in Kenya [8, 12]. These KIM studies covered similar impairments/health conditions as in this Niger study, although they did not include albinism and emotional distress. A KIM study in Bangladesh found a prevalence of 9 per 1000 (95% CI: 8.7–9.4 per 1000) for children under 18, but the study focused on visual, hearing and physical impairments only.

Estimates of prevalence using the KIM method are generally slightly lower than in household surveys using other methodologies for assessing disability. For example, the Washington Group-UNICEF module on Child Functioning used in this study as a preliminary screening tool has been used on its own to generate estimates of disability in population-based surveys. Prevalence of disability (using the cut-off of “a lot of difficulty” in at least one domain) in children 2–17 has been estimated at 2.6% (1.8–3.6%) in north-western Cameroon [35], 2.3% (1.4–3.7%) in Telangana state, India [35], 3.3% (2.6–4.3%) in the Maldives [36] and 4.7 (4.0–5.4%) in Guatemala [29]. In children 5–17 years, prevalence has been measured as 1.5 (1.0–1.7%) in Vanuatu [37] and 1.5% (1.0–2.1%) in Nepal [38], which covers a similar age range as in this study and are similar to the estimates produced from this KIM. In contrast to KIMs, these other studies focus only on functioning and do not include data on the presence of clinical impairments, which can be important for planning health services. Previous studies also indicate that people with self-reported functional difficulties do not always necessarily have underlying clinical impairment [39].

This study also involved new innovations to the KIM. Other KIMs have used screening camps with specialists (e.g. physiotherapists, ophthalmologists, ENT doctors), while this study used paediatricians who visited KI identified children in or near their homes. This change resulted in a significant improvement in response rates, as in this study 85.6% of children identified by KIs were successfully examined by paediatricians, compared to 48% follow-up for screening camps [8]. However, paediatricians faced more challenges in undertaking certain assessments (e.g. visual and hearing impairment in children with intellectual impairments) or providing diagnoses for some conditions. Future KIMs may wish trial combinations of the two strategies, with paediatricians performing the initial assessment but with referral pathways in place to assess more complex cases (e.g. to neurologists and other specialists for evaluations of some intellectual impairments). Further research to better explore the underlying causes of impairments is also needed.

The results of this study carry implications for the design and delivery of services. In this study, many children with disabilities had never sought medical attention for their impairment/health condition and amongst those that did, few found the services to be helpful. Some of the causes of impairments were preventable or correctable—such as impacted ear wax, refractive error or cataracts – while the impact of others on children’s health and functioning could have been reduced with access to timely services. In Niger and many other countries, there are severe shortages of health and social services required for the management of the impairments [40]. For example, there are only 20 ophthalmologists (1.0 per million population) in all of Niger (well below the World Health Organization’s recommended minimum of 4.0 per million population) [41], and 0.02 child psychiatrists per 100,000 population [42]. Across low- and middle-income countries, there are also significant gaps in the availability of and access to rehabilitation providers, medications for epilepsy and psychiatric conditions, specialists in disability-related health services (e.g. audiologists, occupational therapists) and assistive products, and the limited services available tend to be heavily centralised [43, 44]. Further, there is a need for broader inclusive planning to support the participation of children with disabilities. For example, there are severe shortages of inclusive education specialists and resources globally, which contributes to the high proportion of children with disabilities out of school or excluded from the learning process while in school [45, 46]. The lack of access to needed health services and devices, combined with the lack of disability-inclusive planning (e.g. in infrastructure, schooling, transportation), means that most children with impairments will experience decreased functioning and participation (and thus disability).

### Limitations and areas for further research

Several limitations should be taken into account when interpreting the results of this study.

First, the KIM is designed to identify children with moderate/severe disabilities. Children with milder impairments are not intended to be captured through this method, which should be considered when interpreting and comparing prevalence estimate from this study to others using different methodologies.

Second, even among children with moderate/severe impairments, some underreporting is likely. It is possible that KIs did not identify all eligible in their community (e.g. due to challenges recognising certain impairments, social exclusion limiting awareness of or willingness to report some children with disabilities), which would underestimate prevalence estimates. For example, boys were more likely to be identified by KIs (60% of children listed were male), which may reflect greater visibility of boys in the community and potential underreporting for girls. The prevalence of disability, however, was slightly higher amongst girls compared to boys, which may indicate less specific identification of boys amongst KIs. Still, an assessment of the use of KIs to identify impairments in Bangladesh found they generally have a high specificity [47].

By impairment type, most children with visual and hearing impairments had a profound severity, suggesting underreporting and underestimated prevalence of children with moderate sensory impairments, which has been noted in other KIM studies [14]. It was also not possible to conduct visual and hearing assessments using the available assessment tools in many children with intellectual impairments due to challenges understanding and following the test instructions. However, estimates of visual and hearing impairment were adjusted to account for this non-response. Additionally, using the KIM for measuring emotional distress has not been validated; given that emotional distress is often an “invisible” disability, it is likely that prevalence is an underestimate. Further, KIMs do not involve enumeration of all children in the study area, and so the denominator used to estimate prevalence could differ from the true number of children in the target age range. Given these limitations, the overall and impairment-specific prevalence estimates presented in this paper should be interpreted as minimum likely values.

Finally, paediatricians were not able to provide diagnoses for all impairments, particularly intellectual impairments. Further research involving specialists and more advanced equipment/testing materials (e.g. neurologists, EEGs, MRIs) will be needed to provide definitive diagnoses in many cases, particularly for intellectual impairments. Additionally, this study collected data on the caregiver attributed causes of health conditions/impairments but further research is required to explore in greater depth the causes of impairments (e.g. malnutrition, infections/complications during pregnancy and birth) to identify for pathways for prevention. For example, iodine deficiency is a major cause of intellectual impairment globally and can be prevented through successful salt iodisation programmes [48].

## Conclusion

This study is the first use of the KIM in western Africa to estimate the prevalence of childhood disability. The KIM identified a large number of children with disabilities, many of whom had unmet needs for health services. Increased investment in health, rehabilitation and other services (e.g. inclusive education) are required to support the functioning, well-being and participation of children with disabilities.

## Supplementary Information

**Additional file 1. **

**Additional file 2. **

## Data Availability

The datasets generated and/or analysed during the current study are not publicly available as participants did not give their consent for their data to be uploaded onto a public repository but are available from the corresponding author on reasonable request.
