# Supplementary material for: Childhood disability in rural Niger: a population-based assessment using the Key Informant Method
Source: BMC Pediatr. 2022 Mar 31;22:170. doi: 10.1186/s12887-022-03226-0 (PMC8969242; doi:10.1186/s12887-022-03226-0)
Supplement: Supplementary file 1 — Additional file 1. [file 12887_2022_3226_MOESM1_ESM.docx]

| **Male** | | | |
| --- | --- | --- | --- |
| **Impairment/health condition** | **Number** | **Prevalence per 1000 (95% CI)**^a^ | **Prevalence per 1000 range^b^** |
| Disability (any impairment/health condition) | 327 | 12.9 (11.7- 14.1) | 12.6 – 13.2 |
| Physical impairment | 131 | 5.2 (4.4 - 6.0) | 5.0 – 5.3 |
| Hearing impairment | 74 | 5.6 (4.8 - 6.4) | 5.5 - 5.8 |
| Visual impairment | 23 | 1.3 (0.9 - 1.7) | 1.3 - 1.3 |
| Intellectual impairment | 186 | 7.3 (6.4 - 8.2) | 7.1 - 7.5 |
| PTSD/Emotional distress | 11 | 0.4 (0.2 - 0.6) | 0.4 - 0.5 |
| Epilepsy | 61 | 2.4 (1.9 – 2.9) | 2.3 – 2.5 |
| Albinism | 4 | 0.1 (0.0 - 0.2) | 0.1 - 0.2 |
| Multiple | 127 | 5.0 (4.3 – 5.7) | 4.8 - 5.1 |
|  |  |  |  |
| **Female** | | | |
| **Impairment/health condition** | **Number** | **Prevalence per 1000 (95% CI)**^a^ | **Prevalence per 1000 range^b^** |
| Disability (any impairment/health condition) | 270 | 9.9 (8.9- 10.9) | 9.7 – 10.1 |
| Physical impairment | 122 | 4.5 (3.8 - 5.2) | 4.4 – 4.6 |
| Hearing impairment | 59 | 3.8 (3.1 - 4.5) | 3.7 - 3.9 |
| Visual impairment | 22 | 1.1 (0.7 - 1.5) | 1.1 - 1.1 |
| Intellectual impairment | 152 | 5.6 (4.8 - 6.4) | 5.5 - 5.7 |
| PTSD/Emotional distress | 20 | 0.7 (0.4 - 1.0) | 0.7 - 0.8 |
| Epilepsy | 39 | 1.4 (1.0 – 1.8) | 1.4 – 1.5 |
| Albinism | 3 | 0.1 (0.0 - 0.2) | 0.1 - 0.1 |
| Multiple | 109 | 4.0 (3.3 – 4.7) | 3.9 - 4.1 |

^a^Based on assumption that prevalence of disability and impairments/health conditions was the same in children in the pilot village and in non-responders (overall and for specific screens)

^b^Based on sensitivity analysis assuming the prevalence in the non-responders, pilot village was ±10% of prevalence in children followed-up.
