# Supplementary material for: Childhood disability in rural Niger: a population-based assessment using the Key Informant Method
Source: BMC Pediatr. 2022 Mar 31;22:170. doi: 10.1186/s12887-022-03226-0 (PMC8969242; doi:10.1186/s12887-022-03226-0)
Supplement: Supplementary file 2 — Additional file 2. [file 12887_2022_3226_MOESM2_ESM.docx]

**Table S1: Diagnosis of physical impairments**

| **Diagnosis** | **No. of Diagnosis** | **%** |
| --- | --- | --- |
| *Congenital* | *184* | *35.5* |
| Hand deformity | 8 | 1.5 |
| Upper limb absence (full or partial) | 13 | 2.5 |
| Other upper limb impairment | 6 | 1.2 |
| Dysplasia Hip | 2 | 0.4 |
| Club Foot | 10 | 1.9 |
| Lower limb absence or deformity | 24 | 4.6 |
| Other lower limb impairment | 21 | 4.1 |
| Amniotic bands | 1 | 0.2 |
| Arthrogryposis | 2 | 0.4 |
| Other impairment of upper and lower limbs | 51 | 9.8 |
| Spine deformity | 14 | 2.7 |
| Other impairment of the spine | 2 | 0.4 |
| Cleft lip and palate | 1 | 0.2 |
| Other deformity of face/head/neck | 7 | 1.4 |
| Other congenital | 7 | 1.4 |
| Cause not given | 15 | 2.9 |
| *Acquired non-traumatic* | *90* | *17.4* |
| Joint infection | 12 | 2.3 |
| Limb bone infection | 8 | 1.5 |
| Soft tissue infection | 9 | 1.7 |
| Central nervous system infection | 14 | 2.7 |
| Other infection | 4 | 0.8 |
| Bow Leg | 2 | 0.4 |
| Other Joint Deformity | 2 | 0.4 |
| Cause not given | 39 | 7.5 |
| *Trauma* | *38* | *7.3* |
| Burn Contracture | 2 | 0.4 |
| Fracture Malunion | 8 | 1.5 |
| Head Injury | 1 | 0.2 |
| Chronic Dislocation | 2 | 0.4 |
| Joint Stiffness | 6 | 1.2 |
| Tendon Problem | 7 | 1.4 |
| Muscle Problem | 3 | 0.6 |
| Peripheral Nerve Problem | 6 | 1.2 |
| Other trauma | 1 | 0.2 |
| Cause not given | 2 | 0.4 |
| *Neurological* | *206* | *39.8* |
| Epilepsy | 61 | 11.8 |
| Development Delay | 33 | 6.4 |
| Cerebral Palsy | 26 | 5 |
| Paraplegia | 10 | 1.9 |
| Hemiplegia | 32 | 6.2 |
| Quadriplegia | 10 | 1.9 |
| Peripheral Nerve Palsy | 10 | 1.9 |
| Other neurological | 7 | 1.4 |
| Cause not given | 17 | 3.3 |

Some children had multiple diagnoses. Percentages are calculated based on the total number of diagnoses.
